# The interplay between T helper cells and brain barriers in the pathogenesis of multiple sclerosis

**DOI:** 10.3389/fncel.2023.1101379

**Published:** 2023-02-15

**Authors:** Gabriele Angelini, Alessandro Bani, Gabriela Constantin, Barbara Rossi

**Affiliations:** ^1^Department of Medicine, Section of General Pathology, University of Verona, Verona, Italy; ^2^The Center for Biomedical Computing (CBMC), University of Verona, Verona, Italy

**Keywords:** blood–brain barrier, multiple sclerosis, choroid plexus, T helper cells, neuroinflammation, adhesion molecules, meningeal inflammation

## Abstract

The blood–brain barrier (BBB) and the blood-cerebrospinal fluid barrier (BCSFB) represent two complex structures protecting the central nervous system (CNS) against potentially harmful agents and circulating immune cells. The immunosurveillance of the CNS is governed by immune cells that constantly patrol the BCSFB, whereas during neuroinflammatory disorders, both BBB and BCSFB undergo morphological and functional alterations, promoting leukocyte intravascular adhesion and transmigration from the blood circulation into the CNS. Multiple sclerosis (MS) is the prototype of neuroinflammatory disorders in which peripheral T helper (Th) lymphocytes, particularly Th1 and Th17 cells, infiltrate the CNS and contribute to demyelination and neurodegeneration. Th1 and Th17 cells are considered key players in the pathogenesis of MS and its animal model, experimental autoimmune encephalomyelitis. They can actively interact with CNS borders by complex adhesion mechanisms and secretion of a variety of molecules contributing to barrier dysfunction. In this review, we describe the molecular basis involved in the interactions between Th cells and CNS barriers and discuss the emerging roles of dura mater and arachnoid layer as neuroimmune interfaces contributing to the development of CNS inflammatory diseases.

## 1. Introduction

The central nervous system (CNS) is protected against potentially harmful molecules and circulating immune cells by two distinct biological barriers localized at its borders: the blood–brain barrier (BBB) and the blood-cerebrospinal fluid barrier (BCSFB) (Mastorakos and McGavern, 2019). Under physiological conditions, CNS immunosurveillance is mediated by a low and strictly controlled number of immune cells that constantly patrols the BCSFB by migrating through pial vessels or crossing the choroid plexus (Prinz and Priller, 2017). However, during neuroinflammatory diseases, both BBB and BCSFB undergo morphological and functional alterations, promoting leukocyte migration from the blood stream into the CNS parenchyma and CSF and consequent glial and neuronal dysfunction (Solár et al., 2020; Takata et al., 2021).

Multiple sclerosis (MS) represents the most common chronic inflammatory disorder affecting the CNS, and it is characterized by multifocal perivascular inflammatory infiltrates, gliosis, progressive myelin loss, and axon degeneration (Compston and Coles, 2008). It is one of the most widespread causes of neurological disability in young adults, arising usually between 20 and 40 years of age and affecting women about twice as often as men (Compston and Coles, 2008). Although the etiopathology is still unknown, MS is a complex multifactorial disorder, in which genetic susceptibility and environmental factors, such as infectious agents, play a key role in disease development (Sospedra and Martin, 2005). In most clinical cases (85–90%), the disease starts as a relapsing-remitting disorder (RRMS), characterized by transient episodes of neurologic dysfunction and acute lesions in which inflammation is the main component. Chronic forms of the disease are mainly defined by neurodegeneration and brain atrophy with a minor inflammatory component, and can result from a gradual worsening of the RRMS course [secondary progressive MS (SPMS)] or start from the disease onset as primary progressive MS (PPMS) (Kaskow and Baecher-Allan, 2018; Tillery et al., 2018).

Multiple sclerosis has long been supposed to be mediated by an autoimmune process (Rivers et al., 1933; Kabat et al., 1947; Sospedra and Martin, 2005). A crucial role for CD4+ lymphocytes was suggested by pioneering studies in experimental autoimmune encephalomyelitis (EAE), an animal model of MS (Ben-Nun et al., 1981; Zamvil et al., 1985). The contribution of CD4+ T cells has been further dissected by using the 2D2 transgenic mouse strain, whose T cell receptor repertoire biased toward the myelin oligodendrocyte glycoprotein (MOG) 35–55 antigen spontaneously determines the onset of CNS autoimmunity (Bettelli et al., 2003). The genetic risk related to the major histocompatibility complex (MHC) class II locus (Barcellos et al., 2003; Sawcer et al., 2005), along with the local inflammatory response orchestrated particularly by T helper (Th) 1 and Th17 cells (Kunkl et al., 2020), prompted the scientific community to investigate the specific contribution of activated T cell subpopulations in the pathogenesis of MS. A higher number of different populations of activated T cells was found in the CNS parenchyma, CSF and blood of MS patients compared to control subjects indicating that these cells have the capacity to cross brain borders (Wallström et al., 2000; Arbour et al., 2003; Brucklacher-Waldert et al., 2009; El-Behi et al., 2011). These observations opened numerous research lines using the EAE model to elucidate the mechanisms controlling T cell trafficking into the inflamed CNS.

Pathogenic features, clinical symptoms and disease course are highly variable in MS patients, suggesting that multiple mechanisms can contribute to disease development. Two main hypotheses on the role of immune cells in the etiopathogenesis of MS have been proposed to date. One is the “outside-in” hypothesis supporting the idea that autoreactive CD4+ T lymphocytes activate in peripheral lymphoid organs during infections or other inflammatory reactions, supposedly due to molecular mimicry or bystander activation, and then reach the “naïve” CNS starting local autoimmune responses and neuroinflammation. On the other hand, the “inside-out” hypothesis suggests that the pathological process begins within the CNS, leading to the release of highly antigenic constituents that secondarily promote an autoimmune and neuroinflammatory response in predisposed individuals (Stys et al., 2012; Sen et al., 2020). Dysfunctional brain barriers may represent critical contributors to both scenarios, either promoting the onset of the peripheral immune attack according to the “outside-in” hypothesis or sustaining the “inside-out” pathway by facilitating peripheral recruitment of ancillary proinflammatory and autoimmune cells. Moreover, in both circumstances, the pathological outcome of BBB and BCSFB breakdown contributes to myelin and axonal loss and, consequently, to neurodegeneration and neurological impairment (Engelhardt, 2010).

## 2. BBB modulation during MS: The classical paradigm

The BBB represents a specialized layer of endothelial cells with tight junctions sealing cell-to-cell contacts and regulating the passage of cells and molecules between the blood and the CNS. Together, pericytes, astrocytes, microglial cells, neurons, and endothelial cells contribute to the neurovascular unit, a key anatomical and functional structure for the maintenance of CNS homeostasis (Zenaro et al., 2017; Saint-Pol et al., 2020). BBB dysfunction is considered a pathological hallmark in several inflammatory and neurodegenerative disorders, including MS, since this barrier is one of the gateways to the CNS for circulating leukocytes (Prinz and Priller, 2017). BBB perturbation upon neuroinflammatory disorders is associated with two main processes: (i) alteration of junctional molecules leading to BBB breakdown and vascular leakage, and (ii) endothelial activation with upregulation of adhesion molecules and chemokines driving leukocyte recruitment into the brain parenchyma and thus favoring their subsequent local reactivation. While migration of autoreactive T cells into the CNS is required to mount an autoimmune response, other activated T lymphocytes can infiltrate the CNS regardless of antigen specificity, contributing to the inflammation process (Hickey et al., 1991). In support of these data, it was recently shown that in a passive EAE model most invading CD4+ T cells were not myelin-specific. Particularly, these lymphocytes displayed an antigen-independent, bystander-activated, memory phenotype and contributed to disease pathology by expanding the local production of pro-inflammatory cytokines (Lee et al., 2019).

Magnetic resonance imaging (MRI), using gadolinium (Gd) as a marker of cerebral vascular leakage, is a diagnostic tool and a prognostic evaluator for MS (Filippi, 2000; Steinman, 2001; Leray et al., 2010; Polman et al., 2011). Previous studies have shown that BBB alterations may appear at very early disease stages, preceding active lesion formation, and clinical manifestation (Filippi et al., 1998; Plumb et al., 2002; Soon et al., 2007; Alvarez et al., 2015; Barkauskas et al., 2015). A recent report based on elegant *in vitro* experiments even speculates that an intrinsic BBB malfunctioning could represent an additional pathogenetic mechanism for the development of MS (Nishihara et al., 2022). Further, in RRMS patients, MRI clearly indicates how areas of BBB disruption are topologically heterogenous and coincide with perivascular inflammation and demyelinating lesions during relapses (Miller et al., 1998; Treabă et al., 2014). This suggests that BBB dysfunction is an early feature of MS and may favor T cell transendothelial migration and subsequent immune attack contributing to clinical worsening (Ortiz et al., 2014; Spencer et al., 2018). Moreover, as previously shown in animal models, leukocyte-BBB adhesive interactions during the process of leukocyte extravasation in the CNS may lead to vascular inflammation and further BBB impairment, amplifying neuroinflammation and promoting neuronal damage (Siffrin et al., 2010; Rossi et al., 2011, 2021; Zenaro et al., 2013; Rossi and Constantin, 2016; Spencer et al., 2018).

Multiple sclerosis evolution to neurodegeneration and brain atrophy during chronic forms seems to be less associated to BBB alterations and more to brain-compartmentalized self-sustaining pathological processes (Steinman, 2001; Leray et al., 2010). Indeed, the absence of Gd-enhancing lesions and the paucity of therapeutical responses in the progressive stages of MS suggested that the BBB could regain integrity during disease progression (Coles et al., 1999; Molyneux et al., 2000; Filippi and Rocca, 2005; Anderson et al., 2006; Waxman, 2008; Young et al., 2008), whereas brain-compartmentalized inflammation could be fostered by meningeal lymphoid follicles (Serafini et al., 2004; Meinl et al., 2008; Choi et al., 2012). Nevertheless, several observations, such as cortical deposition of fibrinogen and diffuse tight junction aberrations, argue that BBB dysfunction can still be present in the progressive forms of MS (Leech et al., 2007; Yates et al., 2017).

### 2.1. BBB activation and expression of adhesion molecules

Lymphocyte trafficking into the inflamed tissues represents a critical event in the pathogenesis of autoimmune diseases. Based on *in vitro* and *in vivo* evidence, leukocyte migration through the vascular wall is described as a sequential process including distinct adhesive events: tethering, rolling, chemoattractant-dependent activation of integrins, slow rolling, firm adhesion (also called arrest or sticking), crawling, and diapedesis (Ley et al., 2007; Vestweber, 2015). Specificity and diversity in leukocyte-endothelial cell interactions are generated by different combinations of interchangeable ligand-receptor pairs for each step of the adhesion cascade. The molecular specificity directing leukocytes to the site of inflammation is regulated by adhesion molecules such as selectins, integrins, and members of the immunoglobulin superfamily. A critical step in the transition from leukocyte rolling to arrest is the induction of firm adhesion by chemokines expressed on activated endothelium. Chemokine receptors on the surface of rolling immune cells bind to their cognate ligand, triggering a G protein-dependent signaling that leads to the activation of membrane-expressed β_1_ and β_2_ integrins (Montresor et al., 2012). At the BBB level, leukocyte extravasation follows the standard paradigm of cell migration, and the inhibition of adhesion mechanisms using different therapeutical approaches proved to be beneficial in several models of brain inflammatory conditions (Yednock et al., 1992; Piccio et al., 2002; Constantin, 2008; Rossi et al., 2011; Fabene et al., 2013; Zenaro et al., 2015; Farinazzo et al., 2018).

Adhesion molecules expressed by brain endothelial cells have been studied for three decades for their role in lymphocyte trafficking during EAE and MS. P-selectin is upregulated on CNS vasculature during EAE (Piccio et al., 2002, 2005; Döring et al., 2007), whereas E-selectin was found expressed on autoptic cerebral micro-vessels of MS patients (Washington et al., 1994). In support of these data, *in vitro* studies have shown that P-selectin glycoprotein ligand (PSGL)-1, the major P-selectin ligand, is involved in rolling of CD8+ T cells isolated from MS patients and contributes to the transendothelial migration of MS-derived CD4+ T cells (Battistini et al., 2003; Bahbouhi et al., 2009). Moreover, MRI targeting of P-selectin with iron oxide-conjugated antibodies, to evaluate the modulation of its expression on the endothelium during inflammation, was found useful as a predictive method of EAE activity (Fournier et al., 2017). However, blockade of P- and E-selectin had no therapeutic effect on EAE models (Engelhardt et al., 2005; Döring et al., 2007). Intriguingly, combined blockade of P-selectin and α4 integrins resulted in significantly better clinical outcome than anti-α4 integrin alone in EAE mice, suggesting that selectin blockade may have some therapeutic effect in MS as well (Kerfoot et al., 2006). Together, these data suggest that further studies are needed to better understand the potential pathological role of endothelial selectins in MS.

Cellular adhesion molecules (CAM) belonging to the immunoglobulin superfamily such as vascular CAM-1 (VCAM-1), intercellular CAM-1 (ICAM-1), activated leukocyte CAM (ALCAM), and platelet endothelial CAM-1 (PECAM-1) are upregulated on the CNS vasculature, enhancing leukocyte adhesion and migration to the brain in both EAE and MS (Steffen et al., 1994; Cayrol et al., 2008; Steiner et al., 2010; Greenwood et al., 2011; Wimmer et al., 2019). The involvement of VCAM-1 and ICAM-1 is confirmed by the high expression of very late antigen (VLA)-4 and lymphocyte function-activated antigen (LFA)-1, their main ligands, respectively, on the immune infiltrates populating MS lesions (Cannella and Raine, 1995; Bö et al., 1996). ICAM-1 expression on neuro-vasculature was found to strongly correlate with relapses in RRMS patients, while the blood levels of its soluble form is associated with the degree of BBB impairment and disease activity in MS subjects (Cannella et al., 1990; Hartung et al., 1995). Although VLA-4 can also bind fibronectin with low affinity, its high affinity ligand VCAM-1 is believed to have a central role in encephalitogenic T cell migration into the CNS, as suggested by the strong blocking effect of anti-α4 integrin and anti-VCAM-1 antibodies on EAE development, corroborated by the therapeutic success of Natalizumab in MS patients (Yednock et al., 1992; Baron et al., 1993; Chan and Aruffo, 1993; Steffen et al., 1994; Miller et al., 2003; Belachew et al., 2011). Moreover, ALCAM and its counter-ligand CD6 were correlated to an increased risk of MS development (Wagner et al., 2014), further emphasizing the relevance of CAMs in MS.

Chemokines are fundamental molecules for integrin activation and leukocyte migration into the CNS (Constantin, 2008). C-C motif chemokine ligand 19 (CCL19) and CCL21, two chemokines able to induce integrin activation, were found to be expressed on CNS endothelial cells of post-capillary venules in both healthy and EAE mice, and these data correlated to C-C chemokine receptor type 7 (CCR7) expression on perivascular T cells (Alt et al., 2002). However, cerebral expression of CCL19 and CCL21 was not found in MS subjects, suggesting that different chemokines may be responsible for triggering intravascular integrin activation during human disease (Kivisäkk et al., 2004). Several studies have instead identified a constitutive expression of C-X-C motif chemokine ligand 12 (CXCL12) on BBB endothelial cells in EAE mice and MS patients (Krumbholz et al., 2006; McCandless et al., 2006, 2008). Particularly, a CXCL12 expression gradient was detected along the abluminal BBB surface in healthy animals and during early EAE, as well as in uninflamed regions of the MS brain and in control subjects. However, a preferential luminal localization of CXCL12 was found at EAE peak and in active MS lesions, suggesting a role for this chemokine in integrin activation and intravascular leukocyte adhesion (McCandless et al., 2006, 2008). Indeed, in both MS and EAE, the intravascular expression of CXCL12 was associated to the presence of intraluminal CXCR4+ adhering leukocytes as well as to CXCR4 expression on perivascular infiltrating leukocytes (McCandless et al., 2008). Previous studies have also suggested an involvement of CXCR4 in the confinement of infiltrated T cells at the perivascular level, promoting their compartmentalization around blood vessels and preventing widespread parenchymal infiltration (McCandless et al., 2006; Siffrin et al., 2009). Moreover, CXCR7, an alternative receptor for CXCL12, is present on endothelial cells within the CNS and its expression is increased in EAE at sites of inflammatory infiltration (Cruz-Orengo et al., 2011). Notably, CXCR7 was shown to be critical in mediating CXCL12 internalization and redistribution at CNS endothelial barriers and CXCR7 inhibition ameliorated EAE and reduced leukocyte infiltration into the CNS parenchyma (Cruz-Orengo et al., 2011). CCL2, CCL4, and CCL5 were also found to be expressed on cultured brain endothelial cell suggesting that multiple chemokines may contribute to integrin activation and subsequent arrest in inflamed CNS vessels, even if their expression on the BBB and potential role during *in vivo* CNS inflammation remains to be elucidated (Andjelkovic et al., 1999; Quandt and Dorovini-Zis, 2004).

### 2.2. BBB leakage during neuroinflammation

**FIGURE 1:**
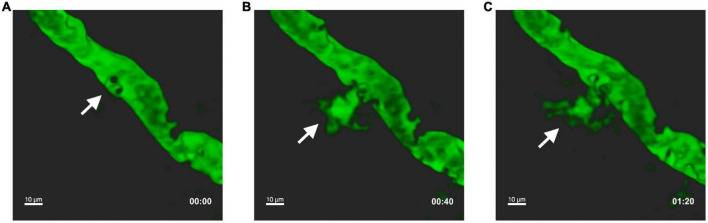
Leukocyte intravascular adhesion contributes to vascular leakage during EAE. *In vivo* two photon laser scanning microscopy was performed on exposed spinal cord of an EAE mouse at disease peak. Pial vessels were visualized through the intravenous injection of 70,000 kDa molecular weight FITC-dextran. White arrows indicate leakage of FITC-dextran into the extravascular space at the point where circulating leukocytes (detected by the imaging system as black dots) perform intravascular adhesion. Representative sequential images at T0 **(A)**, after 40 s **(B)**, and 80 s **(C)**. Adapted and modified from Dusi et al., 2019, (supplementary videos 1 and 2).

During inflammatory conditions, leukocyte adhesion on vascular wall *per se* may lead to increased endothelial permeability (Figure 1). Accordingly, the overexpression of endothelial CAMs during neuroinflammation was shown to promote not only leukocyte adhesion, but also BBB dysfunction and increased permeability. Indeed, ICAM-1 cross-linking leads to reorganization of endothelial cytoskeleton and tight junctions phosphorylation and destabilization, favoring leukocyte adhesion and increasing BBB leakage (Durieu-Trautmann et al., 1994; Adamson et al., 1999). In this context, Rho/ROCK and Rac downstream signals are central modulators of endothelial alterations induced by ICAM-1 and VCAM-1 crosslinking during leukocyte adhesive contacts. Particularly, ICAM-1 engagement elicits RhoA activation and intracellular calcium increase with subsequent reactive oxygen species (ROS) production, actomyosin contraction, and formation of stress fibers (Etienne et al., 1998; Etienne-Manneville et al., 2000; Wang and Doerschuk, 2000). On the other hand, VCAM-1 clustering triggers Rac1 activation and intracellular calcium release, allowing ROS generation, transient disruption of adherent junctions and focal adhesion formation (Lorenzon et al., 1998; Matheny et al., 2000; van Wetering et al., 2003). The relevance of RhoA signaling was confirmed *in vivo*, since its blockade attenuated EAE severity by reducing leukocyte migration into the CNS (Greenwood et al., 2003). This activating signaling within the cerebral endothelium deserves further investigation as a potential molecular target to prevent leukocyte CNS invasion and safeguard BBB integrity in MS. In addition to ICAM-1 and VCAM-1, ALCAM was also found to be present on CNS endothelium during MS and EAE and its expression was associated to T cell migration (Cayrol et al., 2008). However, other studies performed in a chronic EAE model demonstrated that ALCAM maintains BBB integrity by controlling tight junction stability (Lécuyer et al., 2017), suggesting a dual function of ALCAM in neuroinflammation as well as BBB homeostasis (Cayrol et al., 2008; Lyck et al., 2017). Similarly, it was shown that PECAM-1 favors T cell diapedesis across the BBB during neuroinflammation, but also stabilizes BBB integrity (Graesser et al., 2002; Wimmer et al., 2019). Together, these findings suggest that, in addition to the inhibition of leukocyte trafficking into the CNS, BBB stabilization and tight junctions recovery may also represent a therapeutic strategy in brain inflammatory and demyelinating diseases (Spencer et al., 2018).

## 3. T helper cells and BBB breaching: A matter of adhesion molecules and cytokines

### 3.1. Th1 and Th17 cell adhesion to the BBB: A brief overview

Multiple sclerosis has been described as a T cell-mediated autoimmune disease and its widely used model, EAE, is characterized by a Th1 and Th17-driven autoimmune and neuroinflammatory process (Segal, 2019). The study of recruitment kinetics of Th1 and Th17 cells into the CNS during EAE led to the publication of some contrasting reports, probably due to the *in vivo* phenotype plasticity of these cells (Geginat et al., 2014). Whereas some EAE studies indicated Th1 lymphocytes as the earliest CD4+ T cells infiltrating the CNS, followed by secondary Th17 migration (O’Connor et al., 2008), more recent findings suggested that Th17 are the pioneer lymphocytes with a later accumulation of Th1 cells during EAE development (Murphy et al., 2010). Accordingly, *in vitro* migration studies support a preferential capacity of Th17 cells to migrate across the BBB when compared to Th1 lymphocytes (Kebir et al., 2007), but the precise kinetics of Th cell infiltration into the CNS in MS and EAE still remains a matter of debate.

To date, Natalizumab, a monoclonal antibody that blocks activated T cell trafficking targeting the α_4_ chain of VLA-4 integrin, is one of the most efficient treatments for MS (Polman et al., 2006), demonstrating the involvement of VLA-4 in leukocyte migration into the CNS (Schneider-Hohendorf et al., 2014). However, while the blockade of α_4_ integrin completely abolishes Th1 recruitment and partially reduces Th17 cell accumulation in the spinal cord of EAE mice, it fails to prevent Th17 lymphocyte entrance into the cerebrum in an experimental EAE model with atypical brain manifestations (Rothhammer et al., 2011). Under flow adhesion assays using an *in vitro* BBB model confirmed that VCAM-1, the main VLA-4 ligand, is required for the arrest of murine Th1 cells (Steiner et al., 2010). β2 integrins have also a role in firm adhesion and crawling of Th1 lymphocytes on the BBB in *in vitro* models, whereas LFA-1 expression on human Th17 cells has a critical contribution to transmigration across interferon (IFN)-γ activated BBB, suggesting that both VLA-4 and LFA-1 integrins control Th lymphocyte trafficking into the brain in MS (Kebir et al., 2009; Steiner et al., 2010). Intriguingly, in MS patients treated with Natalizumab, Th cells overcome α_4_ blockade by upregulating alternative adhesion receptors, such as PSGL-1 and melanoma CAM (MCAM) (Schneider-Hohendorf et al., 2014). MCAM binds to laminin 411, a component of the endothelial basement membrane, facilitating T lymphocyte penetration into the parenchyma during EAE (Sixt et al., 2001; Wu et al., 2009). Recently, the dual immunoglobulin domain containing CAM (DICAM) was shown to be preferentially expressed on Th17 cells contributing to their migration and pathogenic capacity during EAE. Interestingly, DICAM is particularly expressed on circulating CD4+ T lymphocytes of MS patients and, together with its ligand α_*V*_β_3_, is upregulated on lesion-associated BBB, further suggesting a role for this molecules in Th cell migration during EAE and MS (Du et al., 2016; Charabati et al., 2022).

### 3.2. IFN-γ produced by Th1 cells: Costs and benefits for the BBB

Interferon-γ, the main Th1-related cytokine, is massively produced during neuroinflammatory conditions. The analysis of blood, CSF, and brain tissue samples from MS patients clearly supported the pathogenic role of Th1 cells and their signature cytokine in disease development. Particularly, increased frequency of IFN-γ-producing myelin-reactive T lymphocytes in the blood of MS subjects correlated with the active phase of disease and the worsening of neurological symptoms (Arbour et al., 2003; Moldovan et al., 2003). IFN-γ+ T cells were also found to be enriched in the CSF of MS patients compared to healthy controls (Wallström et al., 2000). Accordingly, IFN-γ levels were significantly elevated in the blood, CSF and CNS lesions of subjects with MS (Mycko et al., 2003; Arellano et al., 2017). The pathogenic effect of IFN-γ is corroborated by an early pilot study evaluating the efficacy of recombinant IFN-γ administration to RRMS patients, who showed a significantly higher exacerbation rate with respect to pre- and post-treatment rates (Panitch et al., 1987).

**FIGURE 2:**
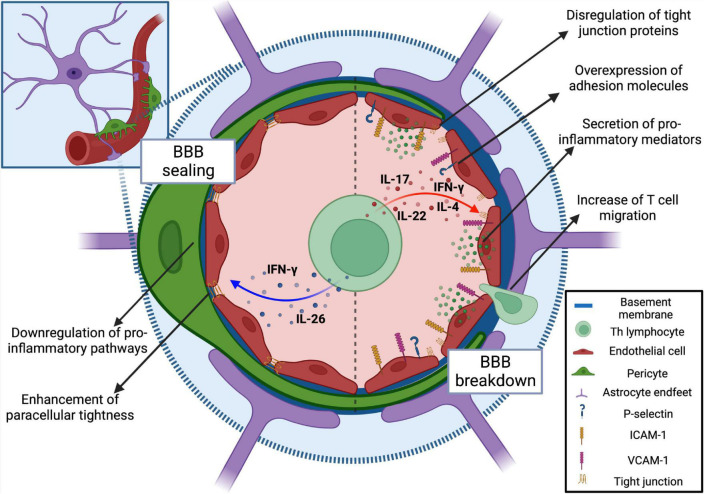
Modulation of BBB permeability and function by T helper cell cytokines. Encephalitogenic T cells communicate with brain vasculature in a paracrine fashion secreting cytokines. During neuroinflammation, IL-17, and IL-22 exert a detrimental effect on brain endothelium functioning. The exposure to these cytokines triggers the loosening of the vascular barrier along with increased expression of adhesion molecules and leukocyte recruitment. Also, although it has been described as an anti-inflammatory cytokine, IL-4 triggers a loss of BBB integrity coupled with changes in endothelial cell morphology. Conversely, IL-26 induces a downregulation of pro-inflammatory and oxidative pathways in endothelial cells, concomitantly stimulating the upregulation of tight junction protein transcripts. IFN-γ can contribute to both BBB breakdown and sealing. Created with BioRender.com.

The overall picture emerging from *in vitro* and *in vivo* observations is that IFN-γ alone is able to regulate the expression of several surface molecules on brain endothelium including MHCI, MHCII, programmed death-ligand (PD-L)1 VCAM-1, mucosal vascular addressin (Mad)CAM-1 and ICAM-1 (Brown et al., 2007; Sonar et al., 2017; Figure 2 and Table 1). Interestingly, time-lapse confocal microscopy experiments showed that IFN-γ, rather than inducing ICAM-1 overexpression, determined a rapid re-localization of this molecule from the basal to the apical side of endothelial cells, potentially promoting leukocyte adhesion (Sonar et al., 2017). Notably, *in vitro* and *in vivo* data suggested that IFN-γ promote BBB leakage by inducing STAT-1 expression and cytoskeleton remodeling affecting tight junction protein organization (Sonar et al., 2017; Bonney et al., 2019). Also, exposure to IFN-γ promotes endothelial permeability through zonula occludens (ZO)-1 and claudin (CLDN) 5 delocalization and adherent junction molecule VE-cadherin perturbation in an *in vitro* system using bEnd3.1 cell line, and these changes were associated to a relevant increase of actin stress fibers and cytoskeletal contraction (Sonar et al., 2017; Liu et al., 2018). *In vitro* inhibition of Rho kinase (ROCK) was sufficient to restore the integrity of brain endothelial cells during IFN-γ treatment by blocking the junctional localization of phosphorylated myosin light chain (Bonney et al., 2019), a phenomenon linked to weakened cell-cell interactions (Hirano and Hirano, 2016). In line with this, *in vivo* ROCK inhibition induced the upregulation of occludin and ZO-1 tight junction proteins on cerebral vessels, making this drug a valuable candidate to tackle BBB breakdown in MS (Yan et al., 2019).

**TABLE 1:** Summary of the effects of Th-derived cytokines on BBB homeostasis.

Cytokine	Main cellular source	Effects on the BBB	Type of effect	References
IFN-γ	Th1	Upregulation of the surface proteins VCAM-1, MadCAM-1, MHC I, and II, PD-L1; apical re-localization of ICAM-1; dysregulation of tight junction proteins ZO-1 and claudin 5; increase of actin stress fibers.	Detrimental	Brown et al., 2007; Sonar et al., 2017; Liu et al., 2018; Bonney et al., 2019
IFN-γ	Th1	Claudin 5-dependent enhancement of paracellular tightness; upregulation of tight junction proteins ZO-1 and occludin; reduction of CXCR7 expression on brain microvessels endothelial cells and consequent decrease of CXCL12 internalization.	Beneficial	Cruz-Orengo et al., 2011; Ni et al., 2014
IL-17	Th17	Mainly mediated by IL-17A; dysregulation of tight junction proteins occludin, ZO-1, and claudin 5; downregulation of *Pecam1* gene expression; trigger of ROS-dependent endothelial contraction; upregulation of the adhesion molecule ICAM-1; secretion of inflammatory mediators such as CCL2, CXCL1, IL-6, and IL-8; CXCR7-dependent scavenging of abluminal CXCL12 in turn reducing leukocyte perivascular retention.	Detrimental	Kebir et al., 2007; Huppert et al., 2010; Cruz-Orengo et al., 2011; Wojkowska et al., 2017; Rahman et al., 2018; Setiadi et al., 2019
IL-22	Th17	Increase of BBB permeability by unknown molecular mechanisms; secretion of CCL2.	Detrimental	Kebir et al., 2007
IL-26	Th17	Downregulation of pro-inflammatory and oxidative phosphorylation pathways; upregulation of *Tjp1*, *Ocln*, and *Cldn18* gene expression.	Beneficial	Broux et al., 2020
IL-4	Th2	Loss of BBB integrity; induction of endothelial cells morphological changes.	Detrimental	Smyth et al., 2018

However, the detrimental effect of IFN-γ on BBB integrity was challenged by Ni et al. (2014), whose findings suggested a role for IFN-γ in inducing the clinching of brain endothelial junctions. In particular, these authors showed that IFN-γ treatment of bEnd3.1 cells enhances their paracellular tightness in a dose-dependent manner and dramatically reduces splenocyte transmigration, with CLDN 5 being essential to link cytokine-dependent signaling to junctional strengthening (Ni et al., 2014). Additionally, exposure of murine brain endothelial cells to IFN-γ decreased CXCR7 expression and led to a reduction of CXCL12 internalization (Cruz-Orengo et al., 2011). Interestingly, blocking CXCL12 sequestration may allow CXCL12 to tether T cells to the perivascular space, preventing infiltration and having a beneficial effect on EAE (Cruz-Orengo et al., 2011). In support of these results, EAE induction in mice expressing IFN-γ receptor exclusively on endothelial cells was sufficient to significantly mitigate brain inflammation, resulting in a lower incidence of atypical symptoms, compared to IFN-γ receptor deficient animals (Ni et al., 2014). Notably, studies performed almost three decades ago showed that IFN-γ signaling plays a protective role during neuroinflammation, as proved by exacerbation of EAE symptoms in IFN-γ^–/–^ mice (Ferber et al., 1996; Willenborg et al., 1996) or disease alleviation upon IFN-γ administration, supporting the idea that IFN-γ promotes BBB stability (Voorthuis et al., 1990; Naves et al., 2013). However, more recent studies showed that IFN-γ signaling is required to trigger spinal cord inflammation and classic EAE, whereas IFN-γ receptor deficiency promotes the development of atypical EAE symptoms, associated to Th17 cell migration in the brainstem and cerebellum (Lees et al., 2008; Stromnes et al., 2008; Pierson et al., 2012). Together, these data suggest that regional CNS responses to IFN-γ, including BBB inflammation and breakdown, determine lesion localization patterns during EAE development.

### 3.3. Th17 cytokines and the BBB: A toxic relationship

IL-17A, IL-17F, IL-22, and IL-26 represent a group of cytokines produced by Th17 cells and are classically associated to the CNS inflammatory milieu during MS (Figure 2 and Table 1). Indeed, increased levels of IL-17 mRNA and IL-17 secreting CD4+ T cells were detected in the blood, CSF, and brain lesions of MS patients (Matusevicius et al., 1999; Lock et al., 2002; Tzartos et al., 2008; Durelli et al., 2009). Particularly, IL-17A is elevated in the CSF of RRMS subjects and correlates with CSF/serum albumin quotient, an index of BBB permeability. In this regard, treating RRMS patients with secukinumab, an anti-IL-17A antibody, reduced MRI lesion activity compared to placebo, supporting a role for IL-17 in disease development (Havrdová et al., 2016).

Cytokines produced by Th17 cells may directly affect BBB integrity and function. Indeed, *in vitro* stimulation of human brain endothelial cells (HBEC) with IL-17A diminishes the expression of tight junction protein-encoding genes *Pecam1*, *Cdh5*, and *Tjp1* and leads to the disassembly of ZO-1 and reduction of occludin and CLDN 5 protein expression (Rahman et al., 2018; Setiadi et al., 2019). Similarly, *in vitro* data obtained using the mouse bEnd.3 cell line showed that IL-17A activates endothelial contractile machinery through a ROS-dependent pathway, inducing occludin downregulation, ZO-1 disorganization, and ICAM-1 upregulation (Huppert et al., 2010) (Figure 2 and Table 1). In line with this, inhibition of IL-17A in a model of EAE led to a reduced leukocyte infiltration of the CNS, lower CNS oxidative stress and milder clinical disease, further demonstrating a role for IL-17 in BBB breakdown (Huppert et al., 2010; Setiadi et al., 2019). Moreover, IL-17, conversely to IFN-γ, induces upregulation of CXCR7 on primary mouse brain endothelial cells (Cruz-Orengo et al., 2011) potentially promoting leukocyte migration, as also suggested by *in vivo* data demonstrating that activation of CXCR7 reduces abluminal CXCL12 concentration and leads to an increased leukocyte entry in the inflamed CNS during EAE (Cruz-Orengo et al., 2011). IL-22 produced by Th17 cells can also affect BBB permeability, as shown by studies performed with primary endothelial cells from human CNS tissue specimens (Kebir et al., 2007; Figure 2 and Table 1). Remarkably, the receptors for IL-17 and IL-22 are absent in CNS samples from healthy human subjects, but they are strongly expressed on CNS endothelial cells within MS lesions, suggesting that the human BBB is highly responsive to the detrimental effect of these two cytokines (Kebir et al., 2007). In agreement with these data, both IL-17 and IL-22 can boost Th cell transmigration across brain endothelium *in vitro*, although IL-17 is more effective in inducing the production of endothelial pro-inflammatory molecules (Kebir et al., 2007; Wojkowska et al., 2017). However, differently from IL-17, which downregulates tight junction proteins, the mechanisms underlying IL-22 effect on the BBB remain uncertain.

Unlike IL-17 and IL-22, IL-26 showed a protective effect on BBB integrity (Broux et al., 2020; Figure 2 and Table 1). Brain endothelial cells treated with IL-26 showed a general downregulation of pro-inflammatory pathways and upregulation of *Tjp1*, *Ocln*, and *Cldn18* gene transcripts. Additionally, IL-26 treatment of EAE mice resulted in a reduced infiltration of pathogenic T lymphocytes and disease attenuation, clearly demonstrating a protective effect of IL-26 at the BBB level (Broux et al., 2020). Overall, the existing literature on MS and EAE point to Th17 cytokines as key players in modulating BBB permeability and leukocyte trafficking during CNS autoimmune inflammatory conditions.

## 4. MS-associated reshaping of BCSFB barrier: The emerging scenario

Whereas the BBB is considered a major gateway for T cell migration from the blood into the CNS, the BCSFB represents a weir tightly regulating the passage of solutes and migrating cells form the blood directly into the CSF. The BCSFB includes two main structures: the leptomeningeal compartment, in which the pial vessels have a central role, and the choroid plexus. The leptomeninges have proven to be an important entry site for peripheral leukocytes (Bartholomäus et al., 2009; Kivisäkk et al., 2009). Notably, a growing body of experimental evidence identifies the leptomeninges as a pivotal anatomical site for T cell reactivation by local antigen presenting cells, development of inflammatory reactions and subsequent CNS invasion by immune cells (Bartholomäus et al., 2009; Kivisäkk et al., 2009; Lodygin et al., 2013; Schläger et al., 2016). On the other hand, the choroid plexus is a highly specialized structure lining the walls of cerebral ventricles and populated by patrolling leukocytes under physiological conditions (de Graaf et al., 2011; Kunis et al., 2013). Interestingly, the selectivity of the choroid plexus barrier is not due to its endothelial cells, which are fenestrated and leaky, but to its innermost epithelial cell layer, which has tight junctions and modulates the trafficking of immune cells into the CSF in response to environmental cues (Maxwell and Pease, 1956; Engelhardt et al., 2001; Ayub et al., 2021).

### 4.1. The leptomeningeal BCSFB

The discovery of lymphoid follicles in the leptomeninges of MS patients and EAE mice redirected the attention from the BBB dysfunction to meningeal inflammation and its potential role in disease pathogenesis (Magliozzi et al., 2004; Serafini et al., 2004). In RRMS and PPMS, the leptomeningeal area seems to be predominantly characterized by a chaotic accumulation of inflammatory cells, whereas in SPMS, immune cells were found in highly organized tertiary lymphoid structures, pointing to leptomeningeal inflammation as a pathogenic driver for disease development (Choi et al., 2012; Howell et al., 2015; Magliozzi et al., 2018). As observed in EAE, MRI investigations corroborated by histopathology studies highlighted a direct correlation between leptomeningeal contrast enhancement, subarachnoid space (SAS)-localized inflammation and subpial cortical pathology in MS patients (Lucchinetti et al., 2011; Choi et al., 2012; Howell et al., 2015; Harrison et al., 2017; Magliozzi et al., 2018; Bergsland et al., 2019; Zurawski et al., 2020). At early stages of human disease, meningeal inflammation is mainly detected in the proximity of areas with altered BBB, cortical demyelination, and gray matter lesions and seems to develop before the emergence of white matter plaques (Lucchinetti et al., 2011). Similarly, inflammatory features appear in the cerebral and spinal cord meninges before EAE onset (Brown and Sawchenko, 2007; Shrestha et al., 2017). Finally, a recent ultra-high field MRI study demonstrated that the cerebral leptomeningeal contrast enhancement magnitude, which reaches a peak of intensity during the acute phase of EAE, is associated to the clinical signs and high inflammatory cell density in EAE mice (Pol et al., 2019). Together, these data underline the importance of the leptomeningeal BCSFB as one of the earliest routes of entry for peripheral immune cells in both MS and EAE.

The SAS is a surgically easily accessible area, and several *in vivo* imaging studies have described the central role of pial vessels during autoreactive T cell recruitment into the CNS (Zenaro et al., 2013; Rossi and Constantin, 2016). Given their permissive endothelial layer and the lack of astrocyte sheathes typical of the BBB, leptomeningeal pial vessels represent a preferential CNS gateway for pioneer circulating autoreactive CD4+ T cells during the preclinical phase of EAE (Bartholomäus et al., 2009). Th cells reach the maximum accumulation in the leptomeninges at EAE peak and migrate in the SAS attracted by the CXCL9-11 and CCL5 chemokines (Bartholomäus et al., 2009; Lodygin et al., 2013; Schläger et al., 2016). The endothelium of inflamed pial vessels expresses P- and E-selectins (Kerfoot and Kubes, 2002; Piccio et al., 2002; Kivisäkk et al., 2003). Particularly, P-selectin was found to be a key molecule involved in leukocyte adhesive interactions in brain pial vessels during the early stages of EAE (Kerfoot and Kubes, 2002; Kerfoot et al., 2006). In agreement with these data, intravital microscopy studies performed in the leptomeningeal microcirculation have shown that a properly glycosylated PSGL-1 is the major P-selectin ligand for efficient tethering and rolling of activated T cells (Piccio et al., 2002, 2005). Likewise, CD8+ T cells from MS patients preferentially exploit PSGL-1 for their rolling on inflamed pial vessels endothelium, further indicating a role for PSGL-1 in activated T cell adhesion in brain pial vessels (Battistini et al., 2003). Interestingly, T cell immunoglobulin and mucin domain 1 (TIM-1) was shown to cooperate with PSGL-1 in the mediation of Th1 and Th17 tethering and rolling on inflamed pial vasculature, contributing to the CNS infiltration of autoreactive T cells and to the development of EAE (Angiari and Constantin, 2014; Angiari et al., 2014). Moreover, integrins VLA-4 and LFA-1 were found to be involved in rolling and G protein-mediated firm adhesion of peripheral leukocytes on inflamed endothelium of cerebral pial vessels, potentially leading to vascular disfunction and leakage (Kerfoot and Kubes, 2002; Piccio et al., 2002; Kerfoot et al., 2006; Figure 1).

Once migrated in the leptomeninges, autoreactive CD4+ T cells are reactivated by local antigen presenting cells and directed to the underlying CNS parenchyma (Bartholomäus et al., 2009; Lodygin et al., 2013; Schläger et al., 2016). However, how autoreactive T cells access the parenchyma from the SAS is still unclear. In this respect, it was suggested that, similar to the blood circulation, CSF dynamics could operate as a physical transport mechanism facilitating cell migration into the inflamed areas of the CNS (Schläger et al., 2016). A potential molecular mechanism controlling T cell migration into the parenchyma could be mediated by laminin 111 (α1β1γ1 or laminin 1), which is preferentially expressed by the leptomeningeal cells and in close contact with astrocyte end feet (Sixt et al., 2001; Agrawal et al., 2006). Also, glia limitans breakdown and microglia activation, both potentially due to meningeal inflammation, may promote autoreactive T cell entry in the cortex and spinal cord during MS and EAE (Gardner et al., 2013; Magliozzi et al., 2018, 2019; James et al., 2020). Furthermore, astrocytes can also contribute to T cell trafficking during EAE. In this context, Th1 and Th17 cytokines induce region-specific astrocyte expression of VCAM-1 and CXCR7, modulating local astrocyte-dependent immune cell trafficking into the CNS during EAE (Williams et al., 2020).

### 4.2. The choroid plexus

**FIGURE 3:**
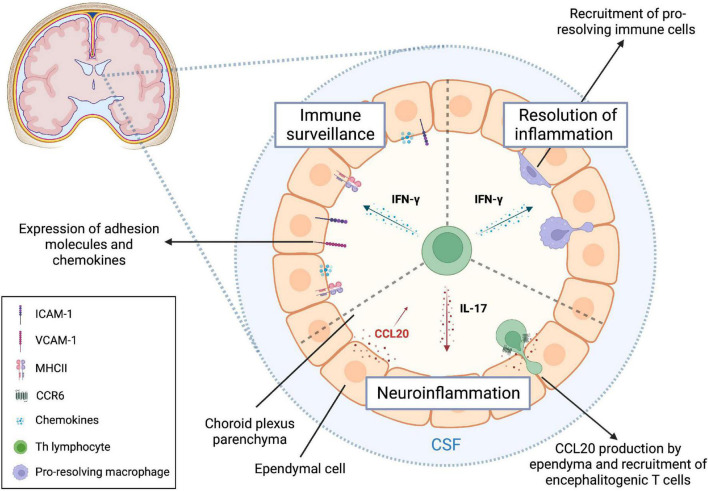
T helper cells regulate choroid plexus function through cytokine secretion. T helper (Th) cells contribute to fine-tune immune processes at the level of the choroid plexus during both homeostasis and neuroinflammation. In the steady state, IFN-γ released by local effector memory CD4+ T lymphocytes is required for an efficient CNS immune surveillance, since it regulates the expression of key trafficking molecules expressed on ependymal cells. Under neuroinflammatory conditions, IFN-γ signaling in the choroid plexus promotes the transmigration of pro-resolving macrophages, which can exert their protective function in the CNS. IL-17 is relevant for EAE development through the induction of CCL20 secretion by choroid plexus epithelium, leading to subsequent invasion of CCR6+ encephalitogenic T lymphocytes. Created with BioRender.com.

Due to its location within the cerebral ventricles and the presence of fenestrated capillaries, the choroid plexus is another important gateway involved in CNS immunosurveillance (Nathanson and Chun, 1989). However, the poor vascular selectivity is compensated by the expression of apical tight junctions and basolateral adherens junctions in the epithelial layer of ependymal cells that strictly controls immune cell trafficking from the choroid plexus stroma into the CSF (Maxwell and Pease, 1956; van Deurs and Koehler, 1979; Lippoldt et al., 2000; Ayub et al., 2021). Indeed, the murine choroid plexus epithelium constitutively expresses ICAM-1, VCAM-1, MadCAM, P-selectin, and CCL20, which may explain, at least in part, why memory CD4+ T cells are the most represented leukocytes within the CSF under physiological conditions (Steffen et al., 1996; Wolburg et al., 1999; Kivisäkk et al., 2003; Reboldi et al., 2009; de Graaf et al., 2011; Figure 3). Additionally, recent *in vitro* studies suggest that, once migrated in the choroid plexus stroma, human CD4+ T cells could further migrate into the CSF by binding ICAM-1 expressed at the luminal side of epithelial cells (Nishihara et al., 2020).

The onset of CNS-targeted autoimmune responses during EAE induces a prominent upregulation of ICAM-1 and VCAM-1 on choroid plexus epithelium, further favoring leukocytes trafficking through this anatomical route (Steffen et al., 1996; Figure 3). When exposed to IFN-γ, the murine choroid plexus epithelium becomes activated, producing chemokines (CCL2, CCL5, CXCL9, CXCL10, CX3CL1, and M-CSF) and upregulating cell adhesion receptors (ICAM-1 and VCAM-1) and MHC-II molecules (Figure 3; Kunis et al., 2013). On the other hand, exposing choroid plexus epithelial cells to IL-17 results in further upregulation of CCL20, a chemokine involved in the recruitment of CCR6+ encephalitogenic Th17 cells during early EAE (Reboldi et al., 2009; Kunis et al., 2013; Figure 3). Notably, CCR6+ CD25- CD4+ pro-inflammatory lymphocytes are present in the CSF of MS patients starting from the earliest disease episode, supporting the pivotal involvement of the evolutionarily conserved CCL20-CCR6 axis in the establishment of a pathological loop amplifying local CNS immune responses (Reboldi et al., 2009; Kara et al., 2015). Interestingly, CD4+ memory T cells expressing CXCR3, a receptor highly present on Th1 cells, were found enriched in the CSF compared to circulating levels (Kivisäkk et al., 2002). However, no differences in CXCR3 expression were detected between MS and control subjects, suggesting a potential role for this chemokine receptor in T cell intrathecal residency (Kivisäkk et al., 2002).

The analysis of postmortem MS brain samples revealed an impairment of epithelial CLDN 3 at the level of the choroid plexus. Accordingly, mice lacking CLDN 3 displayed an earlier EAE onset due to higher levels of infiltrated leukocytes in the CSF (Kooij et al., 2014). The early accumulation of activated T cells in the choroid plexus during EAE suggests a crucial role for this CNS compartment in disease pathogenesis (Brown and Sawchenko, 2007; Murugesan et al., 2012). Throughout the EAE course, the choroid plexus appears enlarged, morphologically altered, and expresses immune-related genes including proinflammatory cytokines, co-stimulatory molecules for T cells and adhesion molecules, suggesting its potential capacity to recall and activate migrating autoreactive T cells (Murugesan et al., 2012). Also, high-throughput studies showed that the majority of T cell receptor repertoire inside the choroid plexus is specific for CNS antigens in mice that were immunized with spinal cord homogenates (Baruch et al., 2013). In addition, recent data demonstrated that the choroid plexus is characterized by the constitutive presence of CNS antigens and antigen presenting cells, that can rapidly react to inflammatory signals, promoting T cell proliferation and a second wave of leukocyte migration into the CNS (Strominger et al., 2018). In agreement with studies performed in mouse models, results obtained on post-mortem choroid plexus samples found increased T cell migration and HLA-DR expression on the choroid plexus stroma of patients with MS compared to control subjects, suggesting that choroid plexus may represent a site for lymphocyte entry in the CSF and antigen presentation also during human disease (Vercellino et al., 2008).

Recent MRI data showed a significant increase of choroid plexus volume in EAE mice compared to untreated animals at baseline, as well as in RRMS patients compared to healthy controls, correlating to acute disease episodes and disability worsening (Fleischer et al., 2021). Notably, Natalizumab treatment proved effective in reducing choroid plexus enlargement in MS patients at follow-up, while dimethyl fumarate, which does not interfere with leukocyte recruitment into the CNS, had no effect (Fleischer et al., 2021). Altogether, these data suggest a role for leukocyte migration through the choroid plexus in promoting MS pathology.

## 5. Dural interface: The outsider

Recent studies showed that the dura mater also represents a bona fide immune interface, opening new scenarios on the role of meningeal immunity during homeostasis and pathological conditions (Rustenhoven et al., 2021). The rediscovery of a full-fledged CSF-draining lymphatic system located in the dura mater sparked interest in the immune properties of this overlooked meningeal structure (Louveau et al., 2015). Interestingly, the ablation of the meningeal lymphatics impairs the drainage of immune cells and macromolecules from the CSF to deep cervical lymph nodes, dampening the capacity of encephalitogenic T cells to mount an efficient autoimmune response during EAE (Louveau et al., 2018). Furthermore, dural sinuses are characterized by VCAM-1 expression and are surrounded by gradients of CXCL12, representing an ideal spot for T cell extravasation (Rustenhoven et al., 2021). The preferential distribution of antigen presenting cells and patrolling T lymphocytes at the peri-sinusal level is also of strategic importance for local immune surveillance, further pointing to this meningeal layer as a true neuro-immune interface (Rustenhoven et al., 2021). Moreover, the abundance of dural dendritic cells with a migratory phenotype suggests an active transport of CNS antigens to the draining lymph nodes (Van Hove et al., 2019). Recent studies have shown that, following EAE induction, the number of dural antigen presenting cells is not altered, but there is a significant peri-sinusal accumulation of peripherally activated MOG-reactive T cells that, once migrated into the dura, acquire a tissue resident memory phenotype (Rustenhoven et al., 2021). However, the role of dural interface during CNS autoimmunity has been recently challenged by data showing that meningeal inflammatory processes underlying MS and EAE pathogenesis take place predominantly in the leptomeningeal compartment, whereas the dura mater is only marginally and passively involved in CNS autoimmunity (Merlini et al., 2022). Interestingly, the pool of dural leukocytes seems to be constantly replenished by the skull and vertebral bone marrow through direct channels under normal conditions, with a massive cell mobilization to the dura upon inflammation (Cugurra et al., 2021; Mazzitelli et al., 2022). However, these newly described immune mechanisms mainly regard myeloid cells, while T lymphocytes seem to migrate preferentially through the blood vessels (Rustenhoven et al., 2021). Overall, the role of dural interface in T cell migration and immune responses during MS and EAE is still unclear and requires further investigation.

## 6. Th2 cells and brain barriers

T helper 2 cells were first described as a distinct subset of IL-4-secreting CD4+ T lymphocytes, laying the foundation for the classical Th1/Th2 dualism (Mosmann et al., 1986). Later, the array of Th2-related cytokines was broadened to also include IL-5, IL-10, and IL-13 (Fiorentino et al., 1989; Fort et al., 2001). Th2 cells and their cytokines are mainly considered neuroprotective in EAE and MS (Weiner et al., 1994; Sospedra and Martin, 2005; Fernando et al., 2014). However, contrasting evidence highlighted that CD4+ T lymphocytes with a Th2-like phenotype clonally expanded and actively fueled the B cell response in MS lesions characterized by antibody and complement deposition (Planas et al., 2015), pointing to a multifaceted role for Th2 cells in CNS autoimmune conditions.

Contrary to Th1 and Th17 cells, whose ability to cross brain barriers has been widely investigated, little is known about Th2 lymphocyte migration into the CNS during neuroinflammatory conditions. Intravital microscopy experiments demonstrated that both murine and human Th2 cells show a reduced capability to adhere in inflamed brain pial vessels compared to Th1 lymphocytes, due to their low expression of a specific glycosylation epitope on PSGL-1 molecule (Wagers et al., 1998; Colantonio et al., 2004; Piccio et al., 2005). However, *in vitro* Th2 cell migration through a layer of HBEC was more efficient when compared to Th1 cells and was dependent on the CCR2-CCL2 axis and ICAM-1 (Biernacki et al., 2001). These *in vitro* data were recently challenged by other studies showing that Th2 lymphocytes display the lowest ability to migrate using *in vitro* models of human BBB and BCSFB, when compared to other Th subsets (Wimmer et al., 2019; Nishihara et al., 2020). These discrepancies suggest that different experimental settings of brain barriers and Th cell preparations may be responsible for the contrasting results obtained using Th2 lymphocytes *in vitro*. Recent findings confirmed a key role for ICAM-1 and also showed a role for CD99 in Th2 cell migration using models of human BBB and BCSFB, suggesting this may also be the case during human neuroinflammatory diseases (Wimmer et al., 2019; Nishihara et al., 2020). Interestingly, recent *in vitro* data showed that ICAM-1 mediate the reverse migration of CSF-derived Th2 and other Th cells through the epithelial layer of the choroid plexus, suggesting that these cells may leave the CNS by crossing the BCSFB (Nishihara et al., 2020).

Despite the seemingly protective role played by Th2 lymphocytes and their signature cytokine, treating *in vitro* cultured HBEC with IL-4 triggers a loss of barrier integrity together with changes in cellular morphology (Smyth et al., 2018; Table 1 and Figure 2). These surprising data indicate that IL-4 and Th2 cell-dependent anti-inflammatory functions are not necessarily linked to protective effects on the BBB, and more studies are needed to clarify the impact of Th2 lymphocytes on brain barriers.

## 7. Perspectives

Whereas T cell migration at the BBB and leptomeningeal level has been more extensively studied, the interplay between T lymphocytes and other CNS borders such as the choroid plexus and dura mater is still largely unexplored. Also, the anatomy of the choroid plexus is still not fully characterized, adding further difficulty to the understanding of this CNS border and its relationship with immune cells (Wolburg and Paulus, 2010). Hopefully, cutting-edge *in vivo* imaging approaches will help to better determine how T cells traffic through the choroid plexus into the CSF (Shipley et al., 2020). A deeper knowledge of the inflammatory events at the level of the choroid plexus may also be relevant from a therapeutic point of view, as suggested by recent studies which propose the targeting of this interface between the systemic circulation and the ventricular system as a novel therapeutic approach for CNS diseases (Bryniarski et al., 2020).

Dura mater has also emerged as an additional neuroimmune interface due to its functional organization and specific localization. However, its involvement in the immune responses and interplay with Th lymphocytes during EAE and MS deserves further investigation. Whether T cells can directly migrate through the meningeal layers is unclear. In support of this possibility, it was shown that CLDN 11, a tight junction protein enriched in the arachnoid mater, is downregulated at later stages of EAE, potentially allowing the migration of T lymphocytes through an impaired arachnoid layer (Uchida et al., 2019). The pathological changes of the arachnoid barrier during CNS autoimmune and inflammatory diseases have been poorly dissected and can constitute a future field of investigation.

Finally, the scientific community has long considered the BBB as a weir, but, at the same time, a major route of leukocyte migration into the brain during neuroinflammation, dedicating significant efforts to the identification of therapeutic targets interfering with Th1 and Th17 cell migration into the brain. Nevertheless, the restoration of BBB morphological integrity and functionality following neuroinflammatory insults may represent a promising therapeutic strategy to accelerate CNS recovery during MS (Spencer et al., 2018).

## Author contributions

All authors listed have made a substantial, direct, and intellectual contribution to the work, and approved it for publication.
